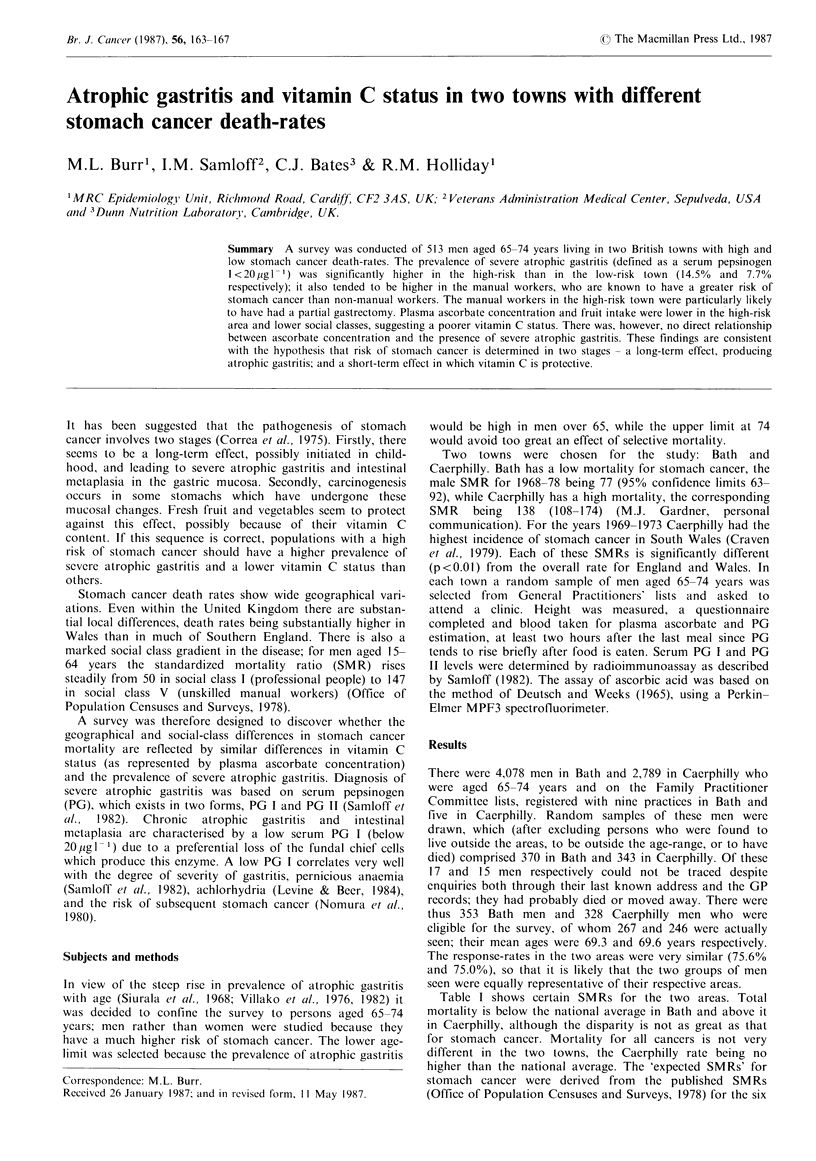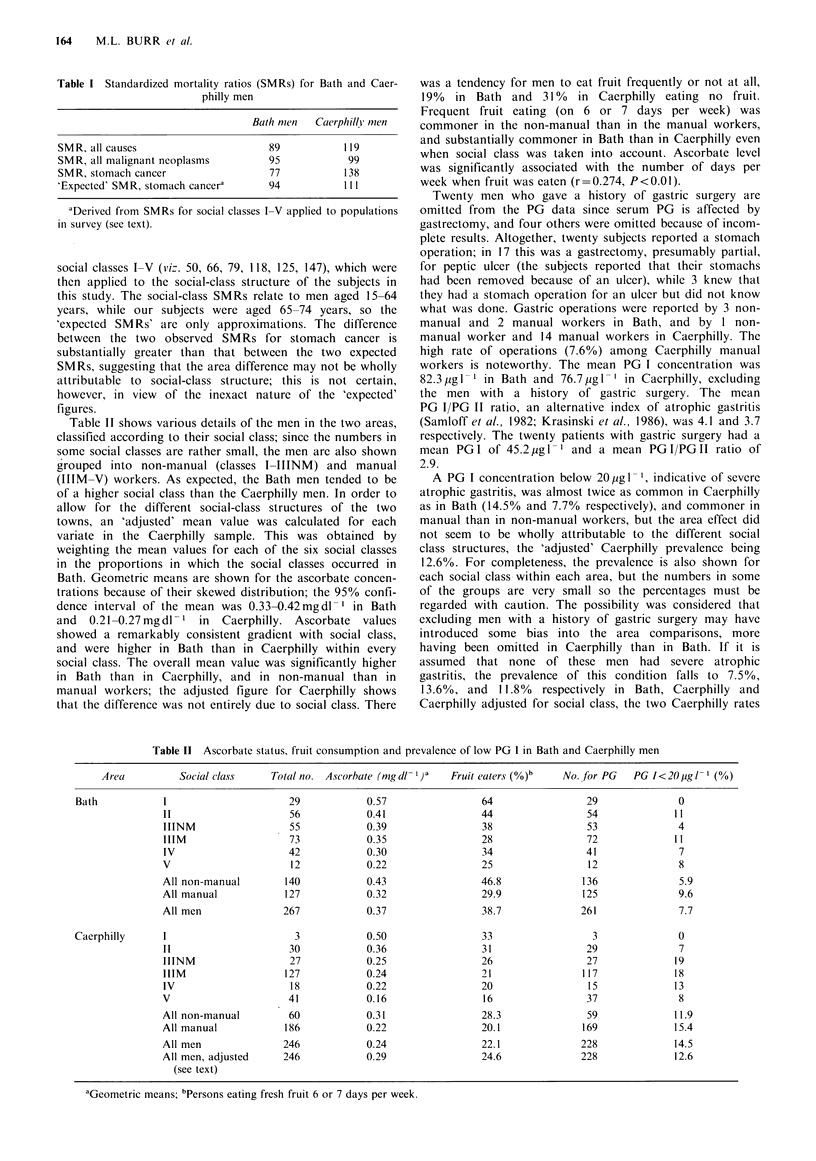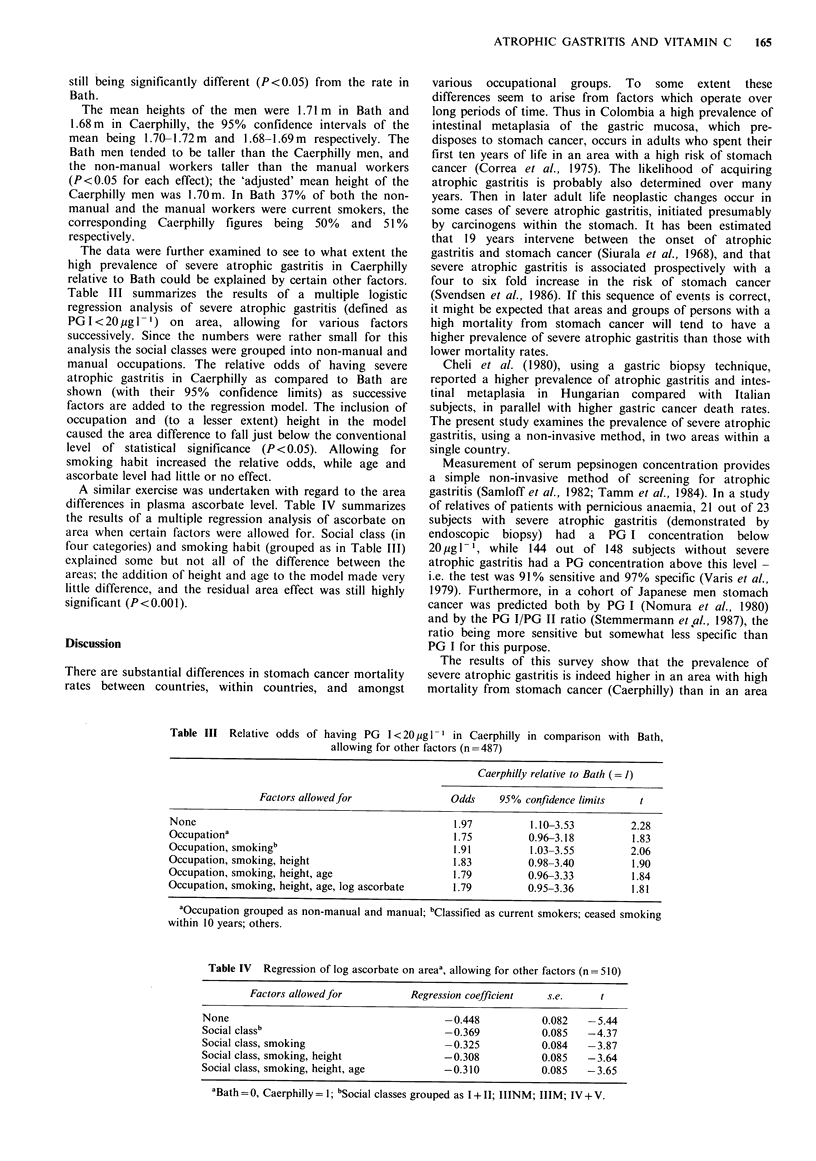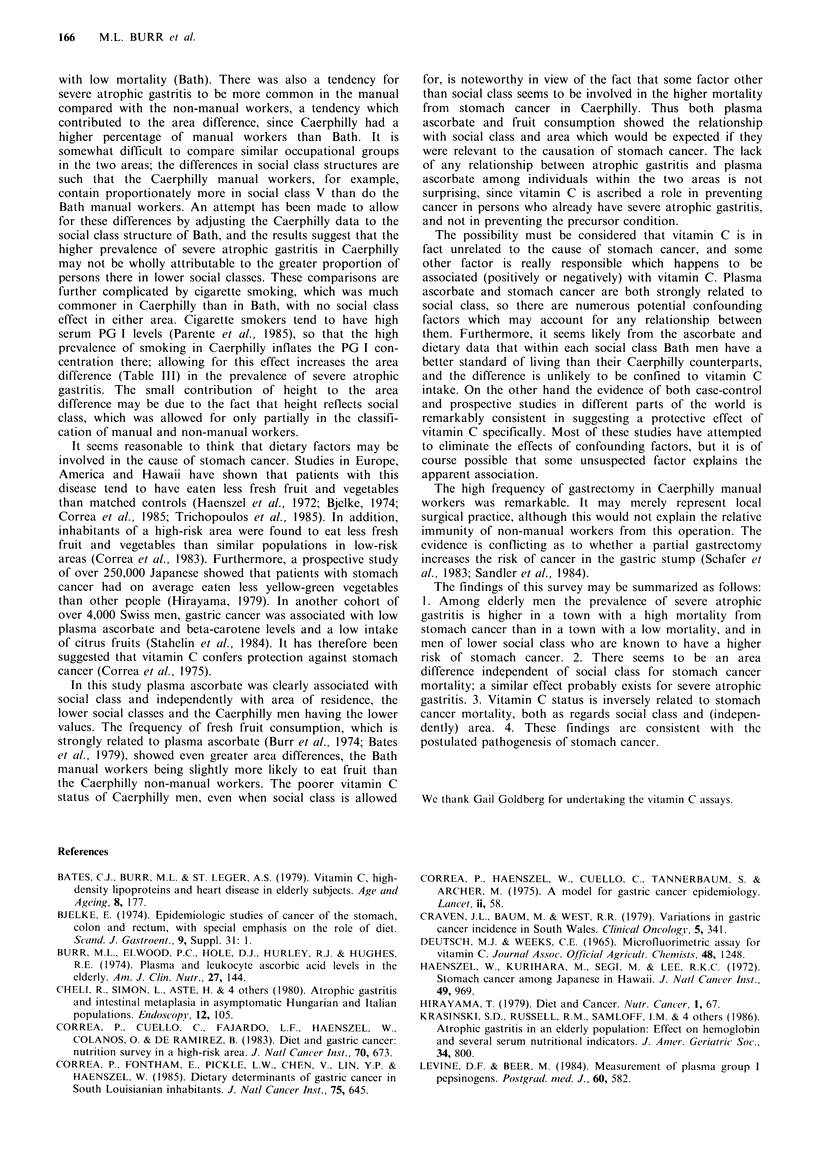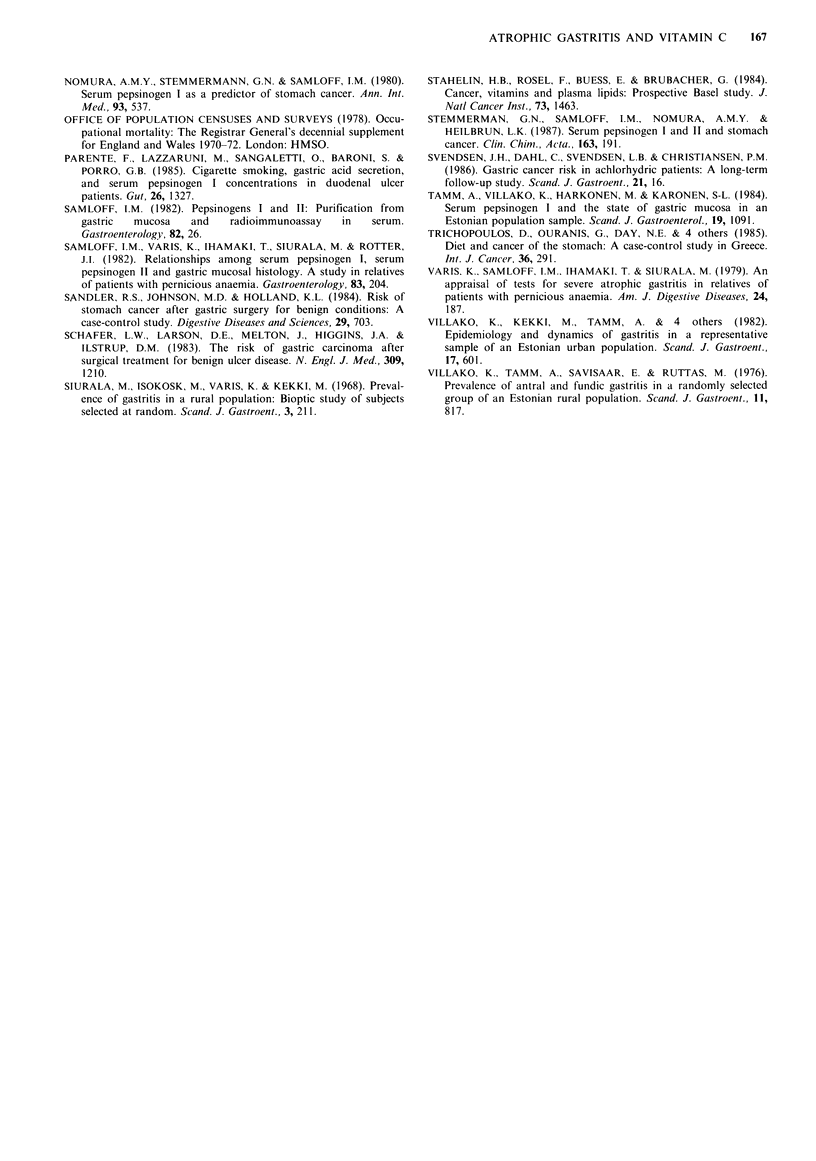# Atrophic gastritis and vitamin C status in two towns with different stomach cancer death-rates.

**DOI:** 10.1038/bjc.1987.178

**Published:** 1987-08

**Authors:** M. L. Burr, I. M. Samloff, C. J. Bates, R. M. Holliday

**Affiliations:** MRC Epidemiology Unit, Cardiff, UK.

## Abstract

A survey was conducted of 513 men aged 65 74 years living in two British towns with high and low stomach cancer death-rates. The prevalence of severe atrophic gastritis (defined as a serum pepsinogen I less than 20 micrograms l-1) was significantly higher in the high-risk than in the low-risk town (14.5% and 7.7% respectively); it also tended to be higher in the manual workers, who are known to have a greater risk of stomach cancer than non-manual workers. The manual workers in the high-risk town were particularly likely to have had a partial gastrectomy. Plasma ascorbate concentration and fruit intake were lower in the high-risk area and lower social classes, suggesting a poorer vitamin C status. There was, however, no direct relationship between ascorbate concentration and the presence of severe atrophic gastritis. These findings are consistent with the hypothesis that risk of stomach cancer is determined in two stages--a long-term effect, producing atrophic gastritis; and a short-term effect in which vitamin C is protective.


Br. J. Cancer (1987), 56, 163–167 © The Macmillan Press Ltd., 1987

### Atrophic gastritis and vitamin C status in two towns with different stomach cancer death-rates

M.L. Burr[1], I.M. Samloff[2], C.J. Bates[3] & R.M. Holliday[1]

[1]*MRC Epidemiology Unit, Richmond Road, Cardiff, CF2 3AS, UK;* [2]*Veterans Administration Medical Center, Sepulveda, USA and* [3]*Dunn Nutrition Laboratory, Cambridge, UK.*

**Summary** A survey was conducted of 513 men aged 65–74 years living in two British towns with high and low stomach cancer death-rates. The prevalence of severe atrophic gastritis (defined as a serum pepsinogen I $<20\,\mu g\,l^{-1}$) was significantly higher in the high-risk than in the low-risk town (14.5% and 7.7% respectively); it also tended to be higher in the manual workers, who are known to have a greater risk of stomach cancer than non-manual workers. The manual workers in the high-risk town were particularly likely to have had a partial gastrectomy. Plasma ascorbate concentration and fruit intake were lower in the high-risk area and lower social classes, suggesting a poorer vitamin C status. There was, however, no direct relationship between ascorbate concentration and the presence of severe atrophic gastritis. These findings are consistent with the hypothesis that risk of stomach cancer is determined in two stages – a long-term effect, producing atrophic gastritis; and a short-term effect in which vitamin C is protective.

It has been suggested that the pathogenesis of stomach cancer involves two stages (Correa *et al.*, 1975). Firstly, there seems to be a long-term effect, possibly initiated in childhood, and leading to severe atrophic gastritis and intestinal metaplasia in the gastric mucosa. Secondly, carcinogenesis occurs in some stomachs which have undergone these mucosal changes. Fresh fruit and vegetables seem to protect against this effect, possibly because of their vitamin C content. If this sequence is correct, populations with a high risk of stomach cancer should have a higher prevalence of severe atrophic gastritis and a lower vitamin C status than others.

Stomach cancer death rates show wide geographical variations. Even within the United Kingdom there are substantial local differences, death rates being substantially higher in Wales than in much of Southern England. There is also a marked social class gradient in the disease; for men aged 15–64 years the standardized mortality ratio (SMR) rises steadily from 50 in social class I (professional people) to 147 in social class V (unskilled manual workers) (Office of Population Censuses and Surveys, 1978).

A survey was therefore designed to discover whether the geographical and social-class differences in stomach cancer mortality are reflected by similar differences in vitamin C status (as represented by plasma ascorbate concentration) and the prevalence of severe atrophic gastritis. Diagnosis of severe atrophic gastritis was based on serum pepsinogen (PG), which exists in two forms, PG I and PG II (Samloff *et al.*, 1982). Chronic atrophic gastritis and intestinal metaplasia are characterised by a low serum PG I (below $20\,\mu g\,l^{-1}$) due to a preferential loss of the fundal chief cells which produce this enzyme. A low PG I correlates very well with the degree of severity of gastritis, pernicious anaemia (Samloff *et al.*, 1982), achlorhydria (Levine & Beer, 1984), and the risk of subsequent stomach cancer (Nomura *et al.*, 1980).

### Subjects and methods

In view of the steep rise in prevalence of atrophic gastritis with age (Siurala *et al.*, 1968; Villako *et al.*, 1976, 1982) it was decided to confine the survey to persons aged 65–74 years; men rather than women were studied because they have a much higher risk of stomach cancer. The lower age-limit was selected because the prevalence of atrophic gastritis would be high in men over 65, while the upper limit at 74 would avoid too great an effect of selective mortality.

Two towns were chosen for the study: Bath and Caerphilly. Bath has a low mortality for stomach cancer, the male SMR for 1968–78 being 77 (95% confidence limits 63–92), while Caerphilly has a high mortality, the corresponding SMR being 138 (108–174) (M.J. Gardner, personal communication). For the years 1969–1973 Caerphilly had the highest incidence of stomach cancer in South Wales (Craven *et al.*, 1979). Each of these SMRs is significantly different ($p<0.01$) from the overall rate for England and Wales. In each town a random sample of men aged 65–74 years was selected from General Practitioners' lists and asked to attend a clinic. Height was measured, a questionnaire completed and blood taken for plasma ascorbate and PG estimation, at least two hours after the last meal since PG tends to rise briefly after food is eaten. Serum PG I and PG II levels were determined by radioimmunoassay as described by Samloff (1982). The assay of ascorbic acid was based on the method of Deutsch and Weeks (1965), using a Perkin-Elmer MPF3 spectrofluorimeter.

### Results

There were 4,078 men in Bath and 2,789 in Caerphilly who were aged 65–74 years and on the Family Practitioner Committee lists, registered with nine practices in Bath and five in Caerphilly. Random samples of these men were drawn, which (after excluding persons who were found to live outside the areas, to be outside the age-range, or to have died) comprised 370 in Bath and 343 in Caerphilly. Of these 17 and 15 men respectively could not be traced despite enquiries both through their last known address and the GP records; they had probably died or moved away. There were thus 353 Bath men and 328 Caerphilly men who were eligible for the survey, of whom 267 and 246 were actually seen; their mean ages were 69.3 and 69.6 years respectively. The response-rates in the two areas were very similar (75.6% and 75.0%), so that it is likely that the two groups of men seen were equally representative of their respective areas.

Table I shows certain SMRs for the two areas. Total mortality is below the national average in Bath and above it in Caerphilly, although the disparity is not as great as that for stomach cancer. Mortality for all cancers is not very different in the two towns, the Caerphilly rate being no higher than the national average. The 'expected SMRs' for stomach cancer were derived from the published SMRs (Office of Population Censuses and Surveys, 1978) for the six

Correspondence: M.L. Burr.
Received 26 January 1987; and in revised form, 11 May 1987.

**Table I** Standardized mortality ratios (SMRs) for Bath and Caerphilly men

| | *Bath men* | *Caerphilly men* |
|---|---|---|
| SMR, all causes | 89 | 119 |
| SMR, all malignant neoplasms | 95 | 99 |
| SMR, stomach cancer | 77 | 138 |
| 'Expected' SMR, stomach cancer[a] | 94 | 111 |

[a]Derived from SMRs for social classes I–V applied to populations in survey (see text).

social classes I–V (*viz.* 50, 66, 79, 118, 125, 147), which were then applied to the social-class structure of the subjects in this study. The social-class SMRs relate to men aged 15–64 years, while our subjects were aged 65–74 years, so the 'expected SMRs' are only approximations. The difference between the two observed SMRs for stomach cancer is substantially greater than that between the two expected SMRs, suggesting that the area difference may not be wholly attributable to social-class structure; this is not certain, however, in view of the inexact nature of the 'expected' figures.

Table II shows various details of the men in the two areas, classified according to their social class; since the numbers in some social classes are rather small, the men are also shown grouped into non-manual (classes I–IIINM) and manual (IIIM–V) workers. As expected, the Bath men tended to be of a higher social class than the Caerphilly men. In order to allow for the different social-class structures of the two towns, an 'adjusted' mean value was calculated for each variate in the Caerphilly sample. This was obtained by weighting the mean values for each of the six social classes in the proportions in which the social classes occurred in Bath. Geometric means are shown for the ascorbate concentrations because of their skewed distribution; the 95% confidence interval of the mean was 0.33–0.42 $mg\,dl^{-1}$ in Bath and 0.21–0.27 $mg\,dl^{-1}$ in Caerphilly. Ascorbate values showed a remarkably consistent gradient with social class, and were higher in Bath than in Caerphilly within every social class. The overall mean value was significantly higher in Bath than in Caerphilly, and in non-manual than in manual workers; the adjusted figure for Caerphilly shows that the difference was not entirely due to social class. There was a tendency for men to eat fruit frequently or not at all, 19% in Bath and 31% in Caerphilly eating no fruit. Frequent fruit eating (on 6 or 7 days per week) was commoner in the non-manual than in the manual workers, and substantially commoner in Bath than in Caerphilly even when social class was taken into account. Ascorbate level was significantly associated with the number of days per week when fruit was eaten ($r=0.274$, $P<0.01$).

Twenty men who gave a history of gastric surgery are omitted from the PG data since serum PG is affected by gastrectomy, and four others were omitted because of incomplete results. Altogether, twenty subjects reported a stomach operation; in 17 this was a gastrectomy, presumably partial, for peptic ulcer (the subjects reported that their stomachs had been removed because of an ulcer), while 3 knew that they had a stomach operation for an ulcer but did not know what was done. Gastric operations were reported by 3 non-manual and 2 manual workers in Bath, and by 1 non-manual worker and 14 manual workers in Caerphilly. The high rate of operations (7.6%) among Caerphilly manual workers is noteworthy. The mean PG I concentration was 82.3 $\mu g\,l^{-1}$ in Bath and 76.7 $\mu g\,l^{-1}$ in Caerphilly, excluding the men with a history of gastric surgery. The mean PG I/PG II ratio, an alternative index of atrophic gastritis (Samloff *et al.*, 1982; Krasinski *et al.*, 1986), was 4.1 and 3.7 respectively. The twenty patients with gastric surgery had a mean PG I of 45.2 $\mu g\,l^{-1}$ and a mean PG I/PG II ratio of 2.9.

A PG I concentration below 20 $\mu g\,l^{-1}$, indicative of severe atrophic gastritis, was almost twice as common in Caerphilly as in Bath (14.5% and 7.7% respectively), and commoner in manual than in non-manual workers, but the area effect did not seem to be wholly attributable to the different social class structures, the 'adjusted' Caerphilly prevalence being 12.6%. For completeness, the prevalence is also shown for each social class within each area, but the numbers in some of the groups are very small so the percentages must be regarded with caution. The possibility was considered that excluding men with a history of gastric surgery may have introduced some bias into the area comparisons, more having been omitted in Caerphilly than in Bath. If it is assumed that none of these men had severe atrophic gastritis, the prevalence of this condition falls to 7.5%, 13.6%, and 11.8% respectively in Bath, Caerphilly and Caerphilly adjusted for social class, the two Caerphilly rates

**Table II** Ascorbate status, fruit consumption and prevalence of low PG I in Bath and Caerphilly men

| *Area* | *Social class* | *Total no.* | *Ascorbate* ($mg\,dl^{-1}$)[a] | *Fruit eaters* (%)[b] | *No. for PG* | *PG I* $<20\,\mu g\,l^{-1}$ (%) |
|---|---|---|---|---|---|---|
| Bath | I | 29 | 0.57 | 64 | 29 | 0 |
| | II | 56 | 0.41 | 44 | 54 | 11 |
| | IIINM | 55 | 0.39 | 38 | 53 | 4 |
| | IIIM | 73 | 0.35 | 28 | 72 | 11 |
| | IV | 42 | 0.30 | 34 | 41 | 7 |
| | V | 12 | 0.22 | 25 | 12 | 8 |
| | All non-manual | 140 | 0.43 | 46.8 | 136 | 5.9 |
| | All manual | 127 | 0.32 | 29.9 | 125 | 9.6 |
| | All men | 267 | 0.37 | 38.7 | 261 | 7.7 |
| Caerphilly | I | 3 | 0.50 | 33 | 3 | 0 |
| | II | 30 | 0.36 | 31 | 29 | 7 |
| | IIINM | 27 | 0.25 | 26 | 27 | 19 |
| | IIIM | 127 | 0.24 | 21 | 117 | 18 |
| | IV | 18 | 0.22 | 20 | 15 | 13 |
| | V | 41 | 0.16 | 16 | 37 | 8 |
| | All non-manual | 60 | 0.31 | 28.3 | 59 | 11.9 |
| | All manual | 186 | 0.22 | 20.1 | 169 | 15.4 |
| | All men | 246 | 0.24 | 22.1 | 228 | 14.5 |
| | All men, adjusted (see text) | 246 | 0.29 | 24.6 | 228 | 12.6 |

[a]Geometric means; [b]Persons eating fresh fruit 6 or 7 days per week.

still being significantly different ($P<0.05$) from the rate in Bath.

The mean heights of the men were 1.71 m in Bath and 1.68 m in Caerphilly, the 95% confidence intervals of the mean being 1.70–1.72 m and 1.68–1.69 m respectively. The Bath men tended to be taller than the Caerphilly men, and the non-manual workers taller than the manual workers ($P<0.05$ for each effect); the 'adjusted' mean height of the Caerphilly men was 1.70 m. In Bath 37% of both the non-manual and the manual workers were current smokers, the corresponding Caerphilly figures being 50% and 51% respectively.

The data were further examined to see to what extent the high prevalence of severe atrophic gastritis in Caerphilly relative to Bath could be explained by certain other factors. Table III summarizes the results of a multiple logistic regression analysis of severe atrophic gastritis (defined as PG I $<20\,\mu g\,l^{-1}$) on area, allowing for various factors successively. Since the numbers were rather small for this analysis the social classes were grouped into non-manual and manual occupations. The relative odds of having severe atrophic gastritis in Caerphilly as compared to Bath are shown (with their 95% confidence limits) as successive factors are added to the regression model. The inclusion of occupation and (to a lesser extent) height in the model caused the area difference to fall just below the conventional level of statistical significance ($P<0.05$). Allowing for smoking habit increased the relative odds, while age and ascorbate level had little or no effect.

A similar exercise was undertaken with regard to the area differences in plasma ascorbate level. Table IV summarizes the results of a multiple regression analysis of ascorbate on area when certain factors were allowed for. Social class (in four categories) and smoking habit (grouped as in Table III) explained some but not all of the difference between the areas; the addition of height and age to the model made very little difference, and the residual area effect was still highly significant ($P<0.001$).

### Discussion

There are substantial differences in stomach cancer mortality rates between countries, within countries, and amongst various occupational groups. To some extent these differences seem to arise from factors which operate over long periods of time. Thus in Colombia a high prevalence of intestinal metaplasia of the gastric mucosa, which predisposes to stomach cancer, occurs in adults who spent their first ten years of life in an area with a high risk of stomach cancer (Correa *et al.*, 1975). The likelihood of acquiring atrophic gastritis is probably also determined over many years. Then in later adult life neoplastic changes occur in some cases of severe atrophic gastritis, initiated presumably by carcinogens within the stomach. It has been estimated that 19 years intervene between the onset of atrophic gastritis and stomach cancer (Siurala *et al.*, 1968), and that severe atrophic gastritis is associated prospectively with a four to six fold increase in the risk of stomach cancer (Svendsen *et al.*, 1986). If this sequence of events is correct, it might be expected that areas and groups of persons with a high mortality from stomach cancer will tend to have a higher prevalence of severe atrophic gastritis than those with lower mortality rates.

Cheli *et al.* (1980), using a gastric biopsy technique, reported a higher prevalence of atrophic gastritis and intestinal metaplasia in Hungarian compared with Italian subjects, in parallel with higher gastric cancer death rates. The present study examines the prevalence of severe atrophic gastritis, using a non-invasive method, in two areas within a single country.

Measurement of serum pepsinogen concentration provides a simple non-invasive method of screening for atrophic gastritis (Samloff *et al.*, 1982; Tamm *et al.*, 1984). In a study of relatives of patients with pernicious anaemia, 21 out of 23 subjects with severe atrophic gastritis (demonstrated by endoscopic biopsy) had a PG I concentration below $20\,\mu g\,l^{-1}$, while 144 out of 148 subjects without severe atrophic gastritis had a PG concentration above this level – i.e. the test was 91% sensitive and 97% specific (Varis *et al.*, 1979). Furthermore, in a cohort of Japanese men stomach cancer was predicted both by PG I (Nomura *et al.*, 1980) and by the PG I/PG II ratio (Stemmermann *et al.*, 1987), the ratio being more sensitive but somewhat less specific than PG I for this purpose.

The results of this survey show that the prevalence of severe atrophic gastritis is indeed higher in an area with high mortality from stomach cancer (Caerphilly) than in an area

**Table III** Relative odds of having PG I$<20\,\mu g\,l^{-1}$ in Caerphilly in comparison with Bath, allowing for other factors (n = 487)

| | *Caerphilly relative to Bath (= 1)* | | |
|---|---|---|---|
| *Factors allowed for* | *Odds* | *95% confidence limits* | *t* |
| None | 1.97 | 1.10–3.53 | 2.28 |
| Occupation[a] | 1.75 | 0.96–3.18 | 1.83 |
| Occupation, smoking[b] | 1.91 | 1.03–3.55 | 2.06 |
| Occupation, smoking, height | 1.83 | 0.98–3.40 | 1.90 |
| Occupation, smoking, height, age | 1.79 | 0.96–3.33 | 1.84 |
| Occupation, smoking, height, age, log ascorbate | 1.79 | 0.95–3.36 | 1.81 |

[a]Occupation grouped as non-manual and manual; [b]Classified as current smokers; ceased smoking within 10 years; others.

**Table IV** Regression of log ascorbate on area[a], allowing for other factors (n = 510)

| *Factors allowed for* | *Regression coefficient* | *s.e.* | *t* |
|---|---|---|---|
| None | −0.448 | 0.082 | −5.44 |
| Social class[b] | −0.369 | 0.085 | −4.37 |
| Social class, smoking | −0.325 | 0.084 | −3.87 |
| Social class, smoking, height | −0.308 | 0.085 | −3.64 |
| Social class, smoking, height, age | −0.310 | 0.085 | −3.65 |

[a]Bath = 0, Caerphilly = 1; [b]Social classes grouped as I + II; IIINM; IIIM; IV + V.

with low mortality (Bath). There was also a tendency for severe atrophic gastritis to be more common in the manual compared with the non-manual workers, a tendency which contributed to the area difference, since Caerphilly had a higher percentage of manual workers than Bath. It is somewhat difficult to compare similar occupational groups in the two areas; the differences in social class structures are such that the Caerphilly manual workers, for example, contain proportionately more in social class V than do the Bath manual workers. An attempt has been made to allow for these differences by adjusting the Caerphilly data to the social class structure of Bath, and the results suggest that the higher prevalence of severe atrophic gastritis in Caerphilly may not be wholly attributable to the greater proportion of persons there in lower social classes. These comparisons are further complicated by cigarette smoking, which was much commoner in Caerphilly than in Bath, with no social class effect in either area. Cigarette smokers tend to have high serum PG I levels (Parente *et al.*, 1985), so that the high prevalence of smoking in Caerphilly inflates the PG I concentration there; allowing for this effect increases the area difference (Table III) in the prevalence of severe atrophic gastritis. The small contribution of height to the area difference may be due to the fact that height reflects social class, which was allowed for only partially in the classification of manual and non-manual workers.

It seems reasonable to think that dietary factors may be involved in the cause of stomach cancer. Studies in Europe, America and Hawaii have shown that patients with this disease tend to have eaten less fresh fruit and vegetables than matched controls (Haenszel *et al.*, 1972; Bjelke, 1974; Correa *et al.*, 1985; Trichopoulos *et al.*, 1985). In addition, inhabitants of a high-risk area were found to eat less fresh fruit and vegetables than similar populations in low-risk areas (Correa *et al.*, 1983). Furthermore, a prospective study of over 250,000 Japanese showed that patients with stomach cancer had on average eaten less yellow-green vegetables than other people (Hirayama, 1979). In another cohort of over 4,000 Swiss men, gastric cancer was associated with low plasma ascorbate and beta-carotene levels and a low intake of citrus fruits (Stahelin *et al.*, 1984). It has therefore been suggested that vitamin C confers protection against stomach cancer (Correa *et al.*, 1975).

In this study plasma ascorbate was clearly associated with social class and independently with area of residence, the lower social classes and the Caerphilly men having the lower values. The frequency of fresh fruit consumption, which is strongly related to plasma ascorbate (Burr *et al.*, 1974; Bates *et al.*, 1979), showed even greater area differences, the Bath manual workers being slightly more likely to eat fruit than the Caerphilly non-manual workers. The poorer vitamin C status of Caerphilly men, even when social class is allowed for, is noteworthy in view of the fact that some factor other than social class seems to be involved in the higher mortality from stomach cancer in Caerphilly. Thus both plasma ascorbate and fruit consumption showed the relationship with social class and area which would be expected if they were relevant to the causation of stomach cancer. The lack of any relationship between atrophic gastritis and plasma ascorbate among individuals within the two areas is not surprising, since vitamin C is ascribed a role in preventing cancer in persons who already have severe atrophic gastritis, and not in preventing the precursor condition.

The possibility must be considered that vitamin C is in fact unrelated to the cause of stomach cancer, and some other factor is really responsible which happens to be associated (positively or negatively) with vitamin C. Plasma ascorbate and stomach cancer are both strongly related to social class, so there are numerous potential confounding factors which may account for any relationship between them. Furthermore, it seems likely from the ascorbate and dietary data that within each social class Bath men have a better standard of living than their Caerphilly counterparts, and the difference is unlikely to be confined to vitamin C intake. On the other hand the evidence of both case-control and prospective studies in different parts of the world is remarkably consistent in suggesting a protective effect of vitamin C specifically. Most of these studies have attempted to eliminate the effects of confounding factors, but it is of course possible that some unsuspected factor explains the apparent association.

The high frequency of gastrectomy in Caerphilly manual workers was remarkable. It may merely represent local surgical practice, although this would not explain the relative immunity of non-manual workers from this operation. The evidence is conflicting as to whether a partial gastrectomy increases the risk of cancer in the gastric stump (Schafer *et al.*, 1983; Sandler *et al.*, 1984).

The findings of this survey may be summarized as follows: 1. Among elderly men the prevalence of severe atrophic gastritis is higher in a town with a high mortality from stomach cancer than in a town with a low mortality, and in men of lower social class who are known to have a higher risk of stomach cancer. 2. There seems to be an area difference independent of social class for stomach cancer mortality; a similar effect probably exists for severe atrophic gastritis. 3. Vitamin C status is inversely related to stomach cancer mortality, both as regards social class and (independently) area. 4. These findings are consistent with the postulated pathogenesis of stomach cancer.

We thank Gail Goldberg for undertaking the vitamin C assays.

### References

BATES, C.J., BURR, M.L. & ST. LEGER, A.S. (1979). Vitamin C, high-density lipoproteins and heart disease in elderly subjects. *Age and Ageing*, **8**, 177.

BJELKE, E. (1974). Epidemiologic studies of cancer of the stomach, colon and rectum, with special emphasis on the role of diet. *Scand. J. Gastroent.*, **9**, Suppl. 31: 1.

BURR, M.L., ELWOOD, P.C., HOLE, D.J., HURLEY, R.J. & HUGHES, R.E. (1974). Plasma and leukocyte ascorbic acid levels in the elderly. *Am. J. Clin. Nutr.*, **27**, 144.

CHELI, R., SIMON, L., ASTE, H. & 4 others (1980). Atrophic gastritis and intestinal metaplasia in asymptomatic Hungarian and Italian populations. *Endoscopy*, **12**, 105.

CORREA, P., CUELLO, C., FAJARDO, L.F., HAENSZEL, W., COLANOS, O. & DE RAMIREZ, B. (1983). Diet and gastric cancer: nutrition survey in a high-risk area. *J. Natl Cancer Inst.*, **70**, 673.

CORREA, P., FONTHAM, E., PICKLE, L.W., CHEN, V., LIN, Y.P. & HAENSZEL, W. (1985). Dietary determinants of gastric cancer in South Louisianian inhabitants. *J. Natl Cancer Inst.*, **75**, 645.

CORREA, P., HAENSZEL, W., CUELLO, C., TANNERBAUM, S. & ARCHER, M. (1975). A model for gastric cancer epidemiology. *Lancet*, **ii**, 58.

CRAVEN, J.L., BAUM, M. & WEST, R.R. (1979). Variations in gastric cancer incidence in South Wales. *Clinical Oncology*, **5**, 341.

DEUTSCH, M.J. & WEEKS, C.E. (1965). Microfluorimetric assay for vitamin C. *Journal Assoc. Official Agricult. Chemists*, **48**, 1248.

HAENSZEL, W., KURIHARA, M., SEGI, M. & LEE, R.K.C. (1972). Stomach cancer among Japanese in Hawaii. *J. Natl Cancer Inst.*, **49**, 969.

HIRAYAMA, T. (1979). Diet and Cancer. *Nutr. Cancer*, **1**, 67.

KRASINSKI, S.D., RUSSELL, R.M., SAMLOFF, I.M. & 4 others (1986). Atrophic gastritis in an elderly population: Effect on hemoglobin and several serum nutritional indicators. *J. Amer. Geriatric Soc.*, **34**, 800.

LEVINE, D.F. & BEER, M. (1984). Measurement of plasma group I pepsinogens. *Postgrad. med. J.*, **60**, 582.

NOMURA, A.M.Y., STEMMERMANN, G.N. & SAMLOFF, I.M. (1980). Serum pepsinogen I as a predictor of stomach cancer. *Ann. Int. Med.*, **93**, 537.

OFFICE OF POPULATION CENSUSES AND SURVEYS (1978). Occupational mortality: The Registrar General's decennial supplement for England and Wales 1970–72. London: HMSO.

PARENTE, F., LAZZARUNI, M., SANGALETTI, O., BARONI, S. & PORRO, G.B. (1985). Cigarette smoking, gastric acid secretion, and serum pepsinogen I concentrations in duodenal ulcer patients. *Gut*, **26**, 1327.

SAMLOFF, I.M. (1982). Pepsinogens I and II: Purification from gastric mucosa and radioimmunoassay in serum. *Gastroenterology*, **82**, 26.

SAMLOFF, I.M., VARIS, K., IHAMAKI, T., SIURALA, M. & ROTTER, J.I. (1982). Relationships among serum pepsinogen I, serum pepsinogen II and gastric mucosal histology. A study in relatives of patients with pernicious anaemia. *Gastroenterology*, **83**, 204.

SANDLER, R.S., JOHNSON, M.D. & HOLLAND, K.L. (1984). Risk of stomach cancer after gastric surgery for benign conditions: A case-control study. *Digestive Diseases and Sciences*, **29**, 703.

SCHAFER, L.W., LARSON, D.E., MELTON, J., HIGGINS, J.A. & ILSTRUP, D.M. (1983). The risk of gastric carcinoma after surgical treatment for benign ulcer disease. *N. Engl. J. Med.*, **309**, 1210.

SIURALA, M., ISOKOSK, M., VARIS, K. & KEKKI, M. (1968). Prevalence of gastritis in a rural population: Bioptic study of subjects selected at random. *Scand. J. Gastroent.*, **3**, 211.

STAHELIN, H.B., ROSEL, F., BUESS, E. & BRUBACHER, G. (1984). Cancer, vitamins and plasma lipids: Prospective Basel study. *J. Natl Cancer Inst.*, **73**, 1463.

STEMMERMAN, G.N., SAMLOFF, I.M., NOMURA, A.M.Y. & HEILBRUN, L.K. (1987). Serum pepsinogen I and II and stomach cancer. *Clin. Chim., Acta.*, **163**, 191.

SVENDSEN, J.H., DAHL, C., SVENDSEN, L.B. & CHRISTIANSEN, P.M. (1986). Gastric cancer risk in achlorhydric patients: A long-term follow-up study. *Scand. J. Gastroent.*, **21**, 16.

TAMM, A., VILLAKO, K., HARKONEN, M. & KARONEN, S-L. (1984). Serum pepsinogen I and the state of gastric mucosa in an Estonian population sample. *Scand. J. Gastroenterol.*, **19**, 1091.

TRICHOPOULOS, D., OURANIS, G., DAY, N.E. & 4 others (1985). Diet and cancer of the stomach: A case-control study in Greece. *Int. J. Cancer*, **36**, 291.

VARIS, K., SAMLOFF, I.M., IHAMAKI, T. & SIURALA, M. (1979). An appraisal of tests for severe atrophic gastritis in relatives of patients with pernicious anaemia. *Am. J. Digestive Diseases*, **24**, 187.

VILLAKO, K., KEKKI, M., TAMM, A. & 4 others (1982). Epidemiology and dynamics of gastritis in a representative sample of an Estonian urban population. *Scand. J. Gastroent.*, **17**, 601.

VILLAKO, K., TAMM, A., SAVISAAR, E. & RUTTAS, M. (1976). Prevalence of antral and fundic gastritis in a randomly selected group of an Estonian rural population. *Scand. J. Gastroent.*, **11**, 817.